# HLA dependent immune escape mechanisms in B-cell lymphomas: Implications for immune checkpoint inhibitor therapy?

**DOI:** 10.1080/2162402X.2017.1295202

**Published:** 2017-03-03

**Authors:** Marcel Nijland, Rianne N. Veenstra, Lydia Visser, Chuanhui Xu, Kushi Kushekhar, Gustaaf W. van Imhoff, Philip M. Kluin, Anke van den Berg, Arjan Diepstra

**Affiliations:** a Department of Hematology, University of Groningen, University Medical Centre Groningen, Groningen, the Netherlands; b Department of Pathology and Medical Biology, University of Groningen, University Medical Centre Groningen, Groningen, the Netherlands

**Keywords:** Diffuse large B-cell lymphoma, Epstein barr virus, human leukocyte antigen, Hodgkin lymphoma, immune checkpoint inhibitor, immune evasion, primary central nervous system lymphoma, major histocompatibility complex, therapy response

## Abstract

Antigen presentation by tumor cells in the context of Human Leukocyte Antigen (HLA) is generally considered to be a prerequisite for effective immune checkpoint inhibitor therapy. We evaluated cell surface HLA class I, HLA class II and cytoplasmic HLA-DM staining by immunohistochemistry (IHC) in 389 classical Hodgkin lymphomas (cHL), 22 nodular lymphocyte predominant Hodgkin lymphomas (NLPHL), 137 diffuse large B-cell lymphomas (DLBCL), 39 primary central nervous system lymphomas (PCNSL) and 19 testicular lymphomas. We describe a novel mechanism of immune escape in which loss of HLA-DM expression results in aberrant membranous invariant chain peptide (CLIP) expression in HLA class II cell surface positive lymphoma cells, preventing presentation of antigenic peptides. In HLA class II positive cases, HLA-DM expression was lost in 49% of cHL, 0% of NLPHL, 14% of DLBCL, 3% of PCNSL and 0% of testicular lymphomas. Considering HLA class I, HLA class II and HLA-DM together, 88% of cHL, 10% of NLPHL, 62% of DLBCL, 77% of PCNSL and 87% of testicular lymphoma cases had abnormal HLA expression patterns. In conclusion, an HLA expression pattern incompatible with normal antigen presentation is common in cHL, DLBCL, PCNSL and testicular lymphoma. Retention of CLIP in HLA class II caused by loss of HLA-DM is a novel immune escape mechanism, especially prevalent in cHL. Aberrant HLA expression should be taken into account when evaluating efficacy of checkpoint inhibitors in B-cell lymphomas.

## Introduction

In the past decade, cancer immunotherapy has made major advances by targeting a series of cell surface molecules known as immune checkpoints. The checkpoint molecules can repress the function of killer and pro-inflammatory lymphocytes. Checkpoint inhibitors are monoclonal antibodies (mAbs) that block these inhibitory receptors, thereby stimulating T-cells and generating an antitumor response.^1-3^ B-cell lymphoma comprise a heterogeneous group of malignancies, which arise from malignant transformation of B-cells. The mAbs against programmed death 1 (PD-1) and cytotoxic T-lymphocyte antigen 4 (CTLA-4) have shown substantial therapeutic activity in heavily treated classical Hodgkin lymphoma (cHL) and encouraging results in relapsed/refractory diffuse large B-cell lymphoma (DLBCL) with overall response rates (ORR) of 87% and 36% respectively.^4-8^ Despite these encouraging results, complete remissions are rare and it remains to be established which patients benefit most from checkpoint inhibition.

Antigen presentation depends on the proper processing of proteins and presentation of peptides through the human leukocyte antigens (HLA).^9^ Normal B-cells present antigens through HLA class I, like any other nucleated cell, and as professional antigen-processing cells also in the context of HLA class II.^10,11^ In cancer cells, so called neo-antigens can arise from proteins that are altered, e.g. by gene mutations. Presentation of these neo-antigens by HLA should induce antitumor immune responses. However, lymphoma cells can prevent these responses by various immune evasive mechanisms, including expression of immune checkpoint molecules.^12,13^ Another mechanism to prevent antitumor immune responses involves loss or aberrant expression of HLA, which precludes presentation of tumor cell specific antigens. Thus, loss or aberrant HLA expression may very well have an impact on the efficacy of checkpoint inhibitors.

Loss of membranous HLA class I and/or HLA class II expression has frequently been described in B-cell lymphomas, including cHL, DLBCL, primary mediastinal B-cell lymphoma (PMBCL) and the immune privileged aggressive B-cell lymphomas of brain and testis.^14-27^ Observations in several B-cell non-Hodgkin lymphomas (NHL) indicate cytoplasmic retention of HLA-molecules in a proportion of patients.^17,20,25,27,28^ In three cHL lymph node cell suspensions, it has been described that disruption of antigen presentation is caused by retention of the class II-associated invariant chain peptide (CLIP) in the membranous HLA class II molecules.^16^ HLA-DM is essential in the intracellular assembly of HLA class II-antigenic peptide complexes. It displaces CLIP from the antigen binding groove of HLA class II molecules, to make this groove accessible for loading of antigens. Lack of HLA-DM results in an apparently normal cell surface expression of HLA class II, but without presentation of antigens and neo-antigens.^9^ In this study, we examined the combined protein expression patterns of HLA class I and HLA class II in a large set of cHL, nodular lymphocyte predominant Hodgkin lymphoma (NLPHL), DLBCL, primary central nervous system lymphoma (PCNSL) and primary testicular lymphoma. We expanded this with HLA-DM as well as CLIP in a subset of patients to determine whether the lymphoma cells apply this alternative mechanism of inducing functional loss of HLA class II antigen presentation.

## Results

### HLA expression in cHL

**Figure 1.**
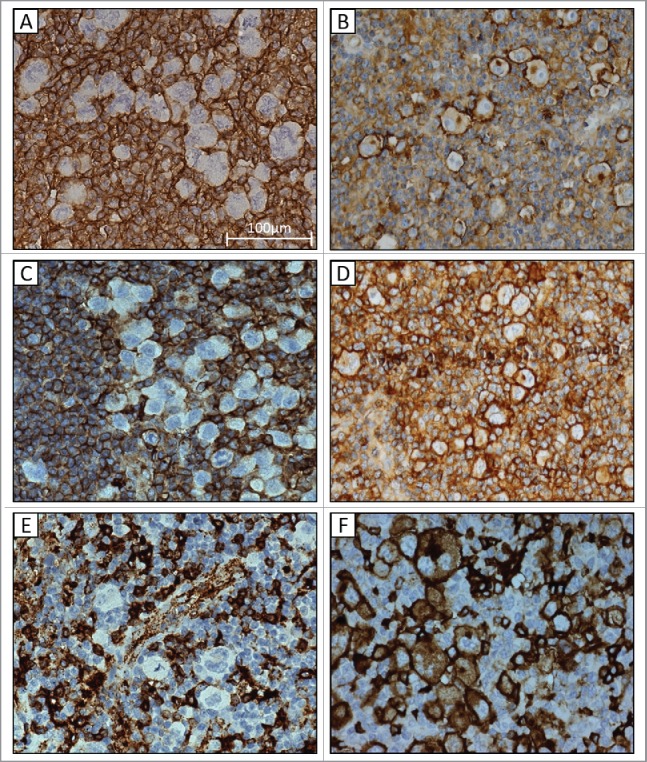
Immunohistochemical staining patterns in formalin fixed paraffin embedded classical Hodgkin lymphoma tissue for HLA class I, B2M and HLA class II. (A and B) negative and positive membranous staining for HLA class I heavy chains. (C and D) negative and positive membranous staining for B2M. (E and F) negative and positive membranous staining for HLA class II. 40x.

Of the 389 cHL samples, 28 were not evaluable for HLA class I and/or HLA class II and were excluded from further analysis. Normal HLA class I cell surface expression was observed in tumor cells in 132 out of 361 cases (36.6%), significantly more often in EBV+cHL (72.6%) than in EBV-cHL (16.8%) (*p* < 0.001). Various aberrant staining patterns were observed, including complete lack of both B2M and HC10 in 52.8% of negative cases (Fig. 1). Of the HLA class I negative cases 38.1% had only cytoplasmic B2M staining and in 8.3% only cytoplasmic HC10 staining. Cell surface expression of HLA class II was present in 214 out of 361 cHL cases (59.3%), significantly more often in EBV+cHL (70.3%) than in EBV-cHL (53.2%) (*p* < 0.001) (Table 1). In 40.1% of the HLA class II cell surface negative cases cytoplasmic staining was observed.

**Table 1.** HLA class I, HLA class II and HLA-DM staining patterns in tumor cells of 361 classical Hodgkin lymphoma patients.

HLA			Hodgkin			
I	II	DM	Total %	EBV+ %	EBV− %	
			(*n* = 361)	(*n* = 128)	(*n* = 233)	*p* value
+	+	+	12.4	27.7	4.1	< 0.01
+	−	ND	11.1	15.6	8.6	0.11
+	+	−	13.0	29.3	4.1	< 0.01
−	+	+	17.6	3.8	25.4	< 0.01
−	−	ND	29.7	14.1	38.2	< 0.01
−	+	−	16.2	9.5	19.6	0.03

ND indicates not done.

### CLIP and HLA-DM expression in cHL

**Figure 2.**
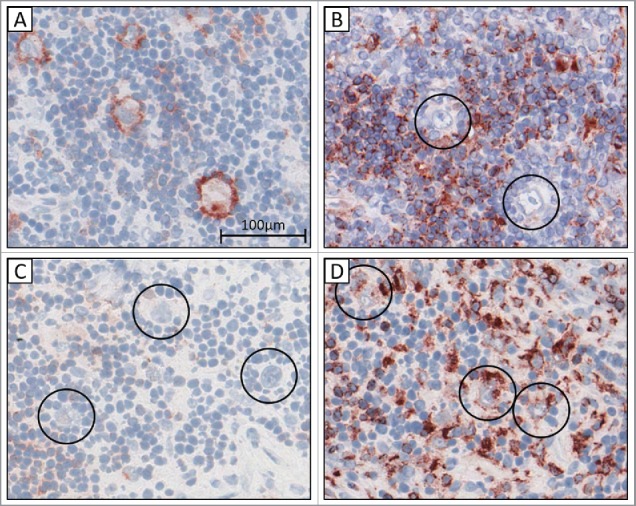
CLIP and HLA-DM immunohistochemistry in frozen classical Hodgkin lymphoma tissue from two representative patients. (A) aberrant membranous CLIP staining in (B) the absence of HLA-DM. (C) normal absence of membranous CLIP staining in (D) presence of cytoplasmic HLA-DM staining. Circles indicate tumor cells. 40x.

Twenty HLA class II membrane positive cases, for which frozen material was available, were stained for CLIP and HLA-DM. CLIP staining was clearly membranous in eight cases, indicating diminishment or lack of presentation of immunogenic peptides in the context of HLA class II. In all of these eight cases HLA-DM staining was completely negative, both in frozen and corresponding formalin-fixed paraffin-embedded (FFPE) tissue. In the 12 cases with normal CLIP staining (weak cytoplasmic), HLA-DM was expressed (Fig. 2). HLA-DM staining was then performed on an additional 69 HLA class II positive FFPE cases. In total, HLA-DM expression was lacking in 44 out of 89 cases of cHL (49.4%).

**Figure 3.**
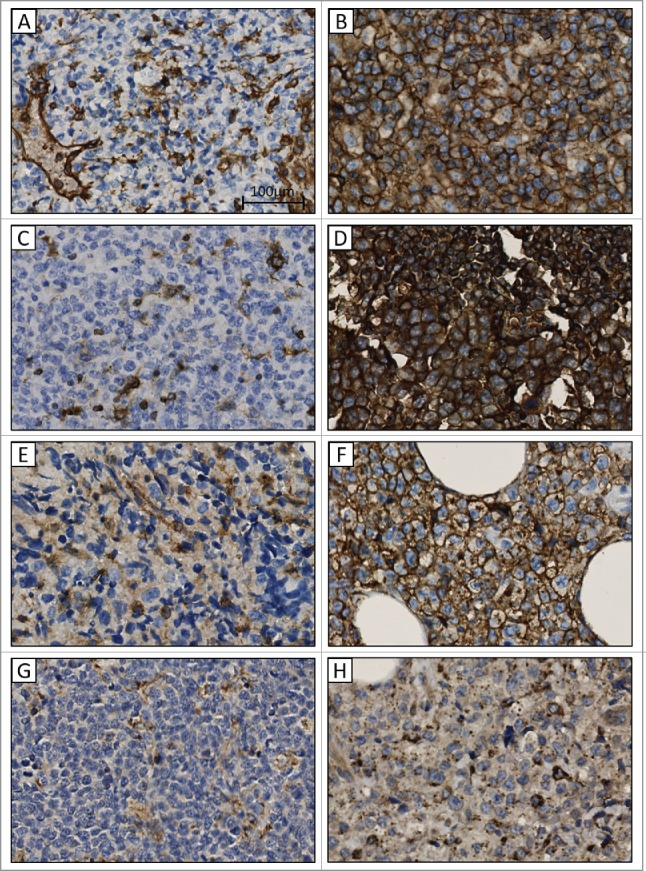
Immunohistochemical staining patterns in formalin fixed paraffin embedded diffuse large B-cell lymphoma tissue for HLA class I, B2M, HLA class II and HLA-DM. (A and B) negative and positive membranous staining for HLA class I heavy chains. (C and D) negative and positive membranous staining for B2M. (E and F) negative and positive membranous staining for HLA class II. (G and H) negative and positive cytoplasmic staining for HLA-DM. 40x.

### HLA expression in NLPHL

Of the 22 NLPHL cases, 1 was not evaluable for HLA class II and was excluded from further analysis. The majority of cases (90.5%) had normal HLA class I and HLA class II expression. Loss of HLA class II was observed in one case and combined loss of HLA class I and class II in another case. None of the HLA class II positive cases had HLA-DM loss.

### HLA expression patterns in DLBCL

Of the 137 DLBCL samples, 20 were not evaluable for either HLA class I, HLA class II or HLA-DM and were excluded from further analysis. HLA class I surface expression was observed in 53 out of 117 cases (45.3%) (Table 2). Of the HLA class I membrane negative cases, two showed cytoplasmic HC10 staining. Cytoplasmic B2M was detected in 31 of the 64 (48.4%) HLA class I negative cases, these did not include the two HC10 cytoplasmic staining cases. Cell surface HLA class II expression was observed in 78 out of 117 DLBCL cases (66.7%). Of the negative membrane cases, cytoplasmic HLA class II expression was seen in 10 out of 39 (25.6%). We found that in the 78 HLA class II cell surface expressing tumor cells, HLA-DM expression was lacking in 11 cases (14.1%). Representative examples of the various staining patterns observed for HC10, B2M and HLA class II are shown in Fig. 2.

**Table 2.** HLA class I, HLA class II and HLA-DM tumor cell staining patterns in 117 diffuse large B-cell lymphoma, 34 primary central nervous system lymphoma and 15 primary testicular lymphoma patients.

HLA			DLBCL%	PCNSL%	Testicular%
I	II	DM	(*n* = 117)	(*n* = 34)	(*n* = 15)
+	+	+	37.6	23.5	13.3
+	−	ND	3.4	5.9	6.7
+	+	−	4.3	0	0
−	+	+	19.7	17.7	0
−	−	ND	29.9	50.0	80
−	+	−	5.1	2.9	0

ND indicates not done.

### HLA-expression patterns in PCNSL and testicular lymphoma

Of the 39 PNCSL samples, 5 were not evaluable for either HLA class I, HLA class II or HLA-DM and were excluded from further analysis. HLA class I and HLA class II cell surface expression was observed in 29.4% of cases, whereas there was a loss of HLA class I and HLA class II in 24 out of 34 (70.6%) and 19 out of 34 (55.9%) cases, respectively (Table 2). HLA-DM loss was observed in 1 case (2.9%). Of the 19 primary testicular lymphoma, 4 were excluded because of missing staining data. Two samples (13.3%) showed normal HLA expression, while 12 samples (80%) lacked both HLA class I and HLA class II (Table 2)

### Combined expression patterns in cHL, NLPHL, DLBCL, PCNSL and testicular lymphoma

Combined results of functional cell surface HLA class I and II expression are presented in Table 3. These combined analyses show that only 12.4% of cHL, 37.6% of DLBCL, 23.5% of PCNSL and 13.3% of testicular lymphoma show an HLA expression pattern that is compatible with normal antigen presentation. In contrast, 90.5% of NLPHL cases show normal HLA class I and HLA class II expression.

**Table 3.** Functional deficits in antigen presentation in tumor cells of classical Hodgkin lymphoma, diffuse large B-cell lymphoma and primary central nervous system lymphoma patients.

Antigen presentation capability	cHL %	NLPHL %	DLBCL %	PCNSL %	Testis %
“Normal”	12.4	90.4	37.6	23.5	13.3
HLA class I dysfunction	17.6	4.8	19.7	17.7	0
HLA class II dysfunction	24.1	0	7.7	5.9	6.7
HLA class I and II dysfunction	45.9	4.8	35.0	52.9	80

*Notes:* Membraneous expression of HLA class I and class II with cytoplasmic HLA-DM. HLA class I dysfunction indicates loss of membraneous HLA class I staining with preserved membraneous HLA class II and cytoplasmic HLA-DM. HLA class II dysfunction refers to loss of HLA class II or loss of HLA-DM with preserved membraneous HLA class I. HLA class I and II dysfunction indicates loss of membraneous HLA class I combined with either loss of membraneous class II or loss of HLA-DM

In EBV-cHL the most prevalent pattern is the combined loss of HLA class I and HLA class II, whereas in EBV+cHL there is more often disruption of HLA class II signaling, either through HLA class II or HLA-DM loss (Table 1).

In DLBCL the most frequent aberrant HLA expression is the loss of both HLA class I and HLA class II (35%). Isolated HLA class I loss (19.7%) is more common than isolated loss of HLA class II and HLA-DM (7.7% and 4.3%) (Tables 2 and 3). The HLA expression patterns were not related to gender, age or stage of the disease (Table S1). In the cohort of patients treated with R-CHOP, we observed no significant difference in progression free survival (PFS) between patients with a normal HLA pattern or aberrant HLA pattern (Log Rank *p* value 0.25) (Fig. S1).

PCNSL and testicular lymphoma have the highest frequency of combined HLA class I and HLA class II loss (52.9% and 80%), with isolated loss of HLA class I or HLA II only being observed incidentally. HLA-DM loss does not seem to constitute a recurrent mechanism for immune escape in either PCNSL or primary testicular lymphoma.

## Discussion

As proper antigen presentation in the context of HLA is expected to be a prerequisite for the action of immune checkpoint inhibitors, we studied HLA expression in retrospective cohorts of B-cell lymphoma. We showed that only a minority of cHL, DLBCL, PNCSL and testicular lymphoma cases show HLA expression that is compatible with normal antigen presentation for both HLA class I and II. Combined HLA class I and HLA class II loss was the most prevalent aberrant pattern in all these lymphoma. In cHL and DLBCL, we identified loss of HLA-DM as a novel mechanism causing disruption of normal antigen presentation in the context of HLA class II.

The minimal requirement for B-cell lymphoma tumor cells to act like professional antigen presenting cells is cell surface expression of the HLA class I heavy chain-B2M complex and the HLA class II heavy chain dimer. Immunohistochemistry (IHC) for these components in normal germinal center B-cells shows strong membranous staining and weak cytoplasmic staining. In EBV-cHL, HLA class I expression has previously been reported to be lacking in 55% (*n* = 38), 81% (*n* = 21) and 71% (*n* = 14) of cases ^29-31^ while, we found 83.2% (*n* = 233). In EBV+cHL these percentages are much lower: 8% (*n* = 25), 24% (*n* = 17), 25% (*n* = 24) and in our series loss of HLA class I was observed in 27.4% (*n* = 128).

In previous publications on DLBCL a wide range of HLA class I loss has been reported, ranging from 34% to 75%,^19-22^ compared with 54.7% in our study. The lower values in this range correspond to studies that made no distinction between membranous and cytoplasmic staining in IHC. Loss of HLA class II expression is consistently less frequently reported than HLA class I loss.^24-27^ Importantly, in the majority of these studies no aberrant cytoplasmic HLA class II staining was described and probably included as positive. In a recent study, specifically looking at cytoplasmic HLA class II expression, aberrant expression patterns were observed in 41%. Interestingly, aberrant HLA class II expression was more often observed in the non-germinal center B-cell type.^28^ Taking this into account, as well as differences in methodology and antibodies used, the results of these studies are compatible with our observed loss of membranous HLA class II expression in 33.3% of cases.

Our IHC results indicate that there is a variety of mechanisms involved in the lack of cell surface HLA expression, judging from different staining patterns in the cytoplasm (absent, diffuse, granular or with Golgi-like localization). In PCNSL and primary testicular lymphoma, loss of HLA-I and HLA-II is frequently due to homozygous or heterozygous deletions of the HLA loci on chromosome 6p21.^18,19^ Both in cHL and DLBCL recent studies indicate that mutations of B2M are a common mechanism for HLA class I loss^20,32,33^ as B2M is required for the stabilization of the HLA class I heavy chain. Decreased HLA class II expression in DLBCL is believed to arise through repression of the HLA locus by decreased expression of CIITA.^27,34-38^ The mechanism behind this repression is unclear. Although CIITA alterations are common in primary mediastinal B-cell lymphoma and result in loss of CIITA^39^ mutations of CIITA in DLBCL are infrequent (0–9%) and can only partially explain the loss of HLA class II.^32,40-43^ In cHL, 15% of cases harbor a translocation of CIITA, resulting in an incomplete downregulation of HLA class II.^44^ In addition, we previously found mutations in 2 out of 6 cHL cell lines.^45^ Whatever the mechanism is, downregulation of HLA is probably a response to continuous antitumor immune responses that increase over time with emergence of antigenic peptides that are related to malignant transformation or disease progression.

Another immune evasive mechanism that we found to be frequent in cHL cases is retained localization of CLIP in the antigen binding groove of HLA class II. In a previous publication it was shown that in three fresh cHL affected lymph node cell suspensions cell surface HLA class II was not occupied by antigenic peptides, but by the non-immunogenic CLIP.^16^ We have now shown that this is caused by lack of expression of HLA-DM. HLA-DO is another HLA accessory molecule that counteracts HLA-DM, but we found no increased expression of HLA-DO (results not shown). Interestingly, presentation of CLIP by dendritic cells antagonizes Th1 polarization. Thus, presentation of CLIP by Hodgkin tumor cells may contribute to the predominant Th2/Treg T-cell populations that are known to directly surround these tumor cells.^46^

Loss of HLA in B-cell lymphoma has been shown to be related to a decrease in number of tumor infiltrating lymphocytes and diminished interferon-gamma responses.^18,21^ Both in cHL and DLBCL lack of membranous HLA class II expression on tumor cells has been shown to be an independent adverse prognostic factor.^15,25,26^ However, with the introduction of rituximab the prognostic value of HLA class II in DLBCL has become less clear.^47^ It was recently suggested that cytoplasmic HLA class II conveys a worse prognosis when compared with HLA class II membrane or HLA class II negative staining.^28^ In our current cohort of DLBCL patients treated with R-CHOP, there was no significant difference in PFS between patients with normal HLA expression and patients with (combined) aberrant expression patterns.

Disrupted antigen presentation is expected to have important implications for the efficacy of checkpoint inhibitors. Despite a high ORR in relapsed cHL treated with a checkpoint inhibitor only 17% of patients achieve a complete remission.^5^ In relapsed DLBCL, checkpoint inhibition as monotherapy shows modest efficacy (ORR of 36%).^6^ However, checkpoint inhibition appears more effective when applied as a consolidation strategy (ORR 51%).^8^ Lack of HLA class I results in loss of presentation of tumor derived neo-antigenic peptides and makes the tumor cells unrecognizable to CD8^+^ cytotoxic T-cells. This implies that the rationale for using immune checkpoint inhibitors might be restricted to HLA class I positive cases. Loss of cell surface expression of HLA class II on the tumor cells may not be a problem, as priming of antitumor immune responses can also occur through professional antigen presenting cells present in the micro-environment. However, in melanoma response to a PD-1 inhibitor does depend on presence of HLA class II on the tumor cells.^48^ Since, aberrant HLA expression is the most prevalent finding that potentially can hamper the efficacy of checkpoint inhibitors, future clinical trials with checkpoint inhibitors should consider HLA expression, both in lymphomas and other cancers.

In conclusion, the majority of cHL, DLBCL, PCNSL and primary testicular lymphoma show HLA expression that is incompatible with normal antigen presentation. The combined losses of HLA class I and HLA class II represent the most frequent mechanism of immune escape. Loss of HLA-DM resulting in the loss of antigen presentation through HLA class II presents a novel mechanism of immune escape in cHL and DLBCL. Our data implicate the importance of taking HLA expression into account when evaluating efficacy of checkpoint inhibitors.

## Materials and methods

### Patients and tumor samples

Primary diagnostic FFPE tissue blocks of 389 cHL, 22 NLPHL, 137 DLBCL not otherwise specified 39 PCNSL and 19 primary testicular lymphoma patients were retrieved from the tissue banks of the pathology department of the University Medical Center Groningen and affiliated hospitals between 1987 and 2011. Part of the HLA expression data in cHL (*n* = 292) was described previously in relation to clinical outcome.^15^ In addition, frozen tissue sections from 20 cHL patients included in the cohort of 389 patients, were included. All cases were reviewed by two experienced hematopathologists. To determine the prognostic value of aberrant HLA expression in DLBCL clinical data on the patients was retrieved from the electronic hospital database of the University Medical Center Groningen. The primary clinical end point was PFS, defined as the time from treatment until relapse or death. Follow-up was completed until December 2015. Patients were treated according to best practice. IHC was performed on anonymized tissue sections in compliance with national ethical guidelines (“Code for Proper Secondary Use of Human Tissue,” Dutch Federation of Medical Scientific Societies) and the declaration of Helsinki.

### Immunohistochemistry and scoring

Tissue sections of 3 μm were cut from formalin fixed and paraffin embedded tissue samples. Immunohistochemical staining was performed according to standard procedures. Briefly, sections were dewaxed with xylene and endogenous peroxidase was blocked. Antigen retrieval was performed in 10 mM Tris (tris-hydroxymethyl-aminomethane)/1 mM EDTA (ethylene diamine tetracetic acid) at pH 9.0. Staining was visualized with mouse mAbs HC10 (HLA class I heavy chains, 1:500, kindly provided by Prof. Dr J. Neefjes, the Netherlands Cancer Institute, Amsterdam), B2M (1:200, DAKO, Glostrup, Denmark), HLA DR/DQ/DP (HLA class II, 1:500, DAKO) and HLA-DM (1:200, BD Biosciences, Breda, the Netherlands). Primary antibodies were detected by secondary and tertiary conjugate antibodies. All cases were stained for B2M, HLA class I and HLA class II. HLA-DM staining was performed in all DLBCL and PCNSL cases and in the cHL cases with membranous HLA class II expression. CLIP staining was performed on fresh frozen material of 20 cHL patients with anti-CLIP (CerCLIP 1:200 BD) and HLA-DM staining with anti HLA-DM (1:200 BD). For all stainings, normal tonsil tissue was used as a positive control. In the cHL cases, EBV status was determined by in situ hybridization using a probe specific for EBV encoded RNAs (EBERs). Scoring was performed by two experienced hematopathologists. Discrepant cases were subsequently discussed until consensus was reached. For HLA class I and HLA class II, membranous staining in the majority of tumor cells was considered normal. Per case, HC10 and B2M were scored consecutively and membranous staining was concordant for all cases. For HLA-DM cytoplasmic staining was considered as normal and for CLIP membranous staining was scored as abnormal.

### Statistical analysis

All categorical variables were expressed as percentages. Where applicable, differences between groups were evaluated by chi-square (for binary variables). A two-tailed *p* value of less than 0.05 indicated statistical significance. PFS curves were estimated according to the Kaplan–Meier method. Between-group differences in PFS were evaluated using the log-rank test. All analyses were performed using IBM SPSS Statistics version 22.

## Supplementary Material

KONI_A_1295202_s02.docx
