# Supplementary material for: HLA dependent immune escape mechanisms in B-cell lymphomas: Implications for immune checkpoint inhibitor therapy?
Source: Oncoimmunology. 2017 Mar 3;6(4):e1295202. doi: 10.1080/2162402X.2017.1295202 (PMC5414870; doi:10.1080/2162402X.2017.1295202)
Supplement: KONI_A_1295202_s02.docx [file koni-06-04-1295202-s001.docx]

| **Supplementary table 1. Clinical characteristics of the 117 patients with diffuse large B-cell lymphoma stained for HLA class I, HLA class II and HLA-DM** | | | | |
| --- | --- | --- | --- | --- |
|  | Total (n = 117) | Normal HLA  (n = 44) | Aberrant HLA  (n = 73) | p-value |
| **Gender** |  |  |  |  |
| Male % (n) | 60.0 (70) | 56.8 (25) | 61.6 (45) | 0.70 |
| Female % (n) | 40.0 (47) | 43.2 (19) | 36.4 (28) |  |
| **Median age** (range) | 65 (15-92) | 63 (15-92) | 66 (19-92) | 0.90 |
| **Ann Arbor** ^#^ |  |  |  |  |
| Stage I/II % (n) | 51.8 (57) | 55.8 (24) | 49.3 (33) | 0.74 |
| Stage III/IV % (n) | 48.2 (53) | 44.2 (19) | 50.7 (34) |  |
| **Localisation** |  |  |  |  |
| Nodal % (n) | 62.4 (73) | 66.0 (29) | 57.1 (44) | 0.61 |
| Extranodal % (n) | 37.6 (44) | 34.0 (15) | 42.9 (33) |  |
| **IPI-score *** |  |  |  |  |
| Low (0-1) % (n) | 46.1 (53) | 45.4 (20) | 46.5 (33) | 0.41 |
| Intermediate (2-3) % (n) | 47.8 (55) | 52.3 (23) | 43.9 (32) |  |
| High (4-5) % (n) | 6.1 (7) | 2.3 (1) | 8.2 (6) |  |
| ^#^ 7 cases missing, * 2 cases missing | | | | |

**Supplementary figure 1**: Progression free survival Kaplan-Meier plot of diffuse large B-cell lymphoma patients treated with R-CHOP, comparing aberrant to normal tumor cell HLA expression.

**Supplementary figure 1.**

**Normal HLA**

**Aberrant HLA**

**
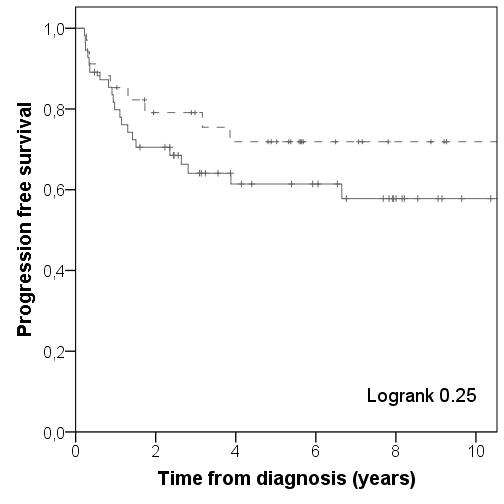
**
